# Ventricular Topology in Congenital Heart Defects Associated with Heterotaxy: Can We Find Patterns Reflecting the Syndrome-Specific Tendency for Visceral Symmetry?

**DOI:** 10.3390/jcdd12110430

**Published:** 2025-10-31

**Authors:** Takhfif Othman, Abdulsalam Mohammad Adnan Alsaiad, Abdulraouf M. Z. Jijeh, Jörg Männer, Talat Mesud Yelbuz

**Affiliations:** 1 King Abdulaziz Cardiac Center, Ministry of National Guard Health Affairs, Riyadh 11426, Saudi Arabia; takhfif.othman@gmail.com (T.O.); alsayadab@mngha.med.sa (A.M.A.A.); jijeha@mngha.med.sa (A.M.Z.J.); 2 King Abdullah International Medical Research Center (KAIMRC), Riyadh 11426, Saudi Arabia; 3 Department of Cardiac Sciences, King Saud Bin Abdulaziz University for Health Sciences, Riyadh 11426, Saudi Arabia; 4 Institute for Anatomy and Cell Biology, UMG, Georg-August-University of Göttingen, D-37075 Göttingen, Germany; jmaenne@gwdg.de

**Keywords:** heterotaxy, congenital heart defects, atrial isomerism, ventricular isomerism, ventricular topology, cardiac looping, translational research

## Abstract

Heterotaxy syndrome is characterized by a tendency for bilaterally symmetric arrangement (isomerism) of inner organs. It is frequently associated with complex congenital heart defects (CHDs). In “heterotaxic” hearts, the tendency for isomerism is confined to the atria. The ventricular segment always shows asymmetric arrangements (D-hand or L-hand topology). This study aimed to determine the statistical distribution of ventricular topology among patients with CHDs associated with heterotaxy and to identify possible associations between ventricular topology and cardiovascular disorders and survival. It is a retrospective cross-sectional study on 192 patients treated at a single center between 2000 and 2023. Our cohort had 115 patients of left atrial isomerism (LAI) and 77 of right atrial isomerism (RAI). The whole cohort (n = 192) showed a bias towards ventricular D-hand topology (67%), which was statistically significant in LAI (74%). In contrast, RAI showed an almost equal distribution (57% D-hand, 43% L-hand). No significant associations were found between ventricular topology and major CHDs or mortality. Significant associations were observed between ventricular topology and cardiac apex position, direction of p-wave axis, and aortic arch sidedness. We conclude that, in the setting of heterotaxy, especially RAI, ventricular topology and aortic arch sidedness both behave as binary anatomical variables showing a tendency for randomized occurrence. This tendency for statistically symmetric distribution is interpreted as reflecting the syndrome-specific tendency for bilateral symmetry.

## 1. Introduction

The outer body shape of humans is characterized by a high degree of bilateral symmetry. This is in contrast to the situation of most inner organs, which normally display bilaterally asymmetric arrangements. Visceral asymmetry is a phylogenetically conserved feature among vertebrates. It evolves from an initially bilateral symmetric situation during early embryogenesis. The emergence of visceral asymmetry is usually described as a three-step process [1,2]. It starts with the breaking of the initial state of bilateral symmetry at a signaling center, called the node, which is located in the body midline of the early embryo. At the subsequent step, left–right (L-R) information is transferred, via molecular signaling cascades, from the midline signaling center to the left- and right-sided organ forming areas where it induces the bilaterally asymmetric expression of body side-specific signaling and transcription factors, such as NODAL and *PITX2*, which define left-sidedness. The paired organ forming areas thereby adopt a molecular left or right identity. At the third step, the molecular L-R identities of the organ forming areas are translated into bilaterally asymmetric morphology.

A bilaterally asymmetric anatomy may not be important for the function of some organs such as the lungs. It is of utmost importance, however, for the normal function of the cardiovascular system of human beings and other lung-breathing vertebrates. Asymmetric anatomy of the heart and great blood vessels dictates the correct alignments and separations of the systemic and pulmonary flow paths at the center of our cardiovascular system [3]. It is therefore no wonder that congenital malformation syndromes with a tendency for bilaterally symmetric patterning of the inner organs—the so-called syndromes of *visceral symmetry* or *visceral isomerism* [4,5]—are frequently associated with complex congenital heart defects (CHDs) such as TOF, DORV, TGA, and TAPVD [4,5,6,7,8].

Congenital syndromes with a tendency for visceral symmetry are nowadays summarized under the diagnostic term *heterotaxy* [9]. Unfortunately, this is a controversial term [10]. Literally, it means “unusual arrangement” and thus may be expected to characterize every arrangement of the inner organs deviating from the usual (situs solitus) one, which would include not only syndromes of *visceral isomerism* but also situs inversus. The present paper is not intended to discuss terminological problems. We, therefore, use the diagnosis *heterotaxy* as suggested by the International Nomenclature Committee for Pediatric and Congenital Heart Disease, which is a diagnosis that does not include situs inversus [9]. Finding the most suitable terminology for these syndromes may come from future discussions among renowned specialists in the field.

It has been noted, already at the time of its first descriptions, that what is currently called heterotaxy syndrome can display either a tendency for bilateral right-sidedness [4] or bilateral left-sidedness [5]. Thus, heterotaxy is typically divided into two subsets. For a long time, these two subsets were not classified according to their well-known tendency for visceral symmetry but according to the status of the spleen [11]. The term *asplenia syndrome* was used for the subset showing a tendency for bilateral right-sidedness and the term *polysplenia syndrome* was used for the subset showing a tendency for bilateral left-sidedness [6]. However, careful examinations of affected patients have shown that the status of the spleen does not regularly correspond to the type of visceral isomerism displayed by affected thoracic organs, especially the type of morphologic symmetry displayed by affected hearts [12]. With regard to the heart, it has been noted that the morphology of the atriums, specifically the anatomy of the atrial appendages (pectinate muscle arrangement and relationship with the other components of the atrial mass), seems to be the best discriminator for the two subsets of the heterotaxy syndrome [12]. This finding has led to the usage of the terms left and right *atrial isomerism* [13] or, more exactly, left and right *isomerism of the atrial appendages* [14,15], instead of the terms asplenia or polysplenia syndromes, although this is not a generally accepted practice [8].

While the atrial heart segment of heterotaxy patients with CHDs can show a strong tendency for morphologic symmetry, there is no evidence of morphologic isomerism of the ventricular segment [16,17,18]. In the setting of atrial appendage isomerism, the two ventricular chambers do not display the same morphology but display different morphologic patterns, which still facilitate identification of the morphologically right and left ventricle, respectively. Moreover, the ventricular heart segment never displays a bilaterally symmetric configuration but usually displays a bilaterally asymmetric (handed) configuration, which can be of either so-called right-hand (normal) or left-hand (invers) topology. Therefore, in the setting of heterotaxy, ventricular topology is usually said to be randomized [19,20].

The situation in hearts with atrial appendage isomerism can be explained by the embryology of the heart [21,22]. The two atrial chambers arise from a common embryonic heart segment that is paired across the body midline. The right-sided atrium is formed by material from the right-sided heart fields and the left-sided atrium is formed by material from the left-sided heart fields. The morphologic identity of the atriums evolves in consequence of the molecular patterning of the embryonic heart-forming fields. In other words, morphologic isomerism of the atriums evolves on the basis of molecular isomerism of the embryonic heart-forming fields and embryonic atriums [23]. The two ventricular heart chambers do not arise from a common embryonic heart segment that is paired across the body midline. They arise from a non-paired midline tube in which the embryonic primordia of the morphological left and right ventricles are initially aligned along the longitudinal (cranio-caudal) axis of the heart tube [24]. Therefore, each ventricular chamber primordium is a midline structure that is formed by materials from the left- as well as right-sided heart fields. The morphological identities of the mature ventricular heart chambers are not determined by the molecular L-R patterning of the embryonic heart fields but by the patterning along the original cranio-caudal heart axis [25]. The ventricular heart chambers are normally brought into their definitive topographical relationships by a morphogenetic process called *ventricular looping*. This process transforms the ventricular segment of the embryonic heart from an initially straight midline tube into a helically coiled tube [24]. Ventricular looping principally can go rightward or leftward and thereby can produce two alternative configurations of the ventricular heart segment, called D-hand and L-hand topology. From these binary anatomical features only the D-hand topology is normally realized during vertebrate embryogenesis, so the spontaneous occurrence of ventricular L-hand topology is normally a very rare event (see Figure 4 in [26]). The normal bias toward the development of ventricular D-hand topology can be regarded as a statistically “asymmetric distribution” of a binary anatomical feature. It suggests that the direction of ventricular looping is determined by the molecular L-R patterning mentioned above.

During the past decades, developmental biologists and clinical geneticists have uncovered numerous genes involved in visceral L-R patterning. Moreover, studies on animal models have shown that visceral asymmetry (visceral situs solitus as well as situs inversus) evolves on the basis of bilaterally asymmetric expression patterns of L-R genes [21], while the tendency for visceral symmetry (heterotaxy) evolves on the basis of bilaterally symmetric expression of left- (molecular left isomerism) or right-sided (molecular right isomerism) genes [1,23]. How the molecular patterning of the embryonic heart controls asymmetric (chiral) looping of its ventricular segment is still an unsolved question. In a rough simplification we can say that, during the past 100 years, two different types of mechanistic concepts have evolved on the basis of experimental data. The first attributes ventricular looping primarily to chiral forces within the ventricular segment. This idea has become a popular hypothesis during the past decade [27,28,29], but its origin can be traced back to the early 1920s. Early experimental data suggest that each heart-forming field has its own intrinsic tendency for helical coiling of the ventricular heart segment. The right for leftward looping and the left for rightward looping [30,31,32,33,34,35]. Early experimental data furthermore indicate that the intrinsic tendency for helical coiling is stronger in the left than the right heart-forming field, suggesting that the looping behavior of the ventricular heart segment is normally dominated by the intrinsic forces of its left half, so that ventricular looping normally produces D-hand topology [34,36]. Recent data attribute the chiral behavior of the embryonic ventricles to intrinsic cell chirality controlled by nodal signaling [28]. The data principally corresponds to the above-mentioned early data in that they show that the two halves of the embryonic ventricle show opposite chiral biases and that one half is dominant [28]. In contrast to the early data, however, it is the right half that is dominant and drives D-looping [27,28,29]. Aside from these puzzling inconsistencies of the data, we can say that, if we assume that the intrinsic tendencies for helical coiling of the two halves of the embryonic ventricular heart segment are determined by molecular L-R patterning of the heart-forming fields, we expect, in cases of molecular isomerism, that ventricular looping should display a subset-dependent statistical distribution, where D-looping is found almost exclusively in one type of molecular isomerism, whereas L-looping is found almost exclusively in the other type of molecular isomerism.

The second type of mechanistic concept postulates that ventricular looping results from the combined action of intrinsic and extrinsic forces [37,38,39]. Intrinsic forces are responsible only for ventral bending of the ventricular tube. The bending ventricular tube is forced to adopt a helical configuration primarily due to external mechanical constraints provided by the pericardial wall. Physical models have indeed shown that the complex helical shape of the ventricular heart loop can evolve on the basis of bilaterally symmetric starting conditions. However, in contrast to the normal situation in vertebrate embryos, ventricular looping simulations under bilaterally symmetric starting conditions lead to a statistically random (50:50) distribution of the two looping phenotypes [40]. This suggest that the molecular L-R asymmetry of the heart-forming fields may not be responsible for the helical deformation of the ventricular segment of the embryonic heart tube but rather cause normal bias toward the development of the D-hand topology phenotype. Simulations have shown that this bias may be attributed to the emergence of subtle morphological asymmetries at the venous or arterial heart pole before looping [40,41] and data from mouse mutants have shown that transient pre-looping activity of nodal at the heart poles is required to amplify and coordinate these subtle asymmetries [42].

We can summarize that, in an individual case of CHD with heterotaxy, the tendency for bilateral symmetry of an affected heart is found only at the level of the atrial segment. The ventricular segment never displays a tendency for bilateral symmetry [16,17,18]. According to embryological concepts, however, we can expect that a tendency for bilateral symmetric development of the ventricular segment may become apparent in the form of statistical distribution patterns of D-hand and L-hand topologies found in large populations of CHD patients with heterotaxy. Depending on the mechanistic concept used to explain the embryology of ventricular topology, we expect to find different patterns. According to the “intrinsic looping only” concept, L-hand topology should tend to occur only in one subset of the syndrome while D-hand topology should tend to occur only in the other subset. In such a scenario, ventricular topology would be subset-dependent and thereby would reflect isomeric molecular patterning of the embryonic ventricular heart segment. According to the intrinsic/extrinsic looping concept, however, there should be a tendency for randomization (50:50) in both subsets of the heterotaxy syndrome. Such a situation can be regarded as a statistically “symmetric distribution” of a binary anatomical feature reflecting the syndrome specific tendency for bilateral symmetry.

The present study was conducted to determine the statistical distribution of ventricular D-hand and L-hand topology in a relatively large group of human patients with CHDs in the setting of heterotaxy. This was primarily performed to check the validity of the two above-mentioned embryological concepts. Since the type of ventricular topology may have influence on the survival of heterotaxy patients, and thereby may mask the initial distribution pattern, we have additionally searched for ventricular topology-specific associations with cardiovascular anomalies.

## 2. Methods

### 2.1. Patient Population

Our study is a retrospective cross-sectional study on a population of 192 patients in which CHDs occurred in the setting of heterotaxy. It was a single center study conducted in a specialized cardiovascular center (King Abdulaziz Cardiac Center [KACC]) located at King Abdulaziz Medical City in Riyadh, Saudi Arabia.

We systematically reviewed imaging data from echocardiography and abdominal ultrasound, advanced imaging modalities such as cardiac and chest computed tomography (CT) and cardiac magnetic resonance imaging (MRI), as well as data from ECG’s 24 h Holter monitoring for all cases of CHDs with heterotaxy that were diagnosed in KACC from January 2000 to March 2023.

The primary diagnosis of CHD with heterotaxy syndrome found in the patient records was mainly based on echocardiography. However, our retrospective diagnosis started with the analysis of image data from CT or MRI as the superior methods for diagnosis of the subsets of left atrial appendage isomerism (LAI) or right atrial appendage isomerism (RAI). Such image data was available for the majority of our patients (n = 108). Only if CT or MRI data was not available (n = 84) did we go to echocardiographic imaging data, which was available for all our patients. However, we did not try to obtain any comparative data between echocardiography and CT/MRI as diagnostic tools since this is beyond the scope of this study. Thus, we were mainly focused on confirming the diagnosis with all available data combined whenever available (CT, MRI, echocardiography, abdominal US, and X-ray). For further details of our heterotaxy diagnosis criteria see Section 2.3 below.

**Inclusion criteria**:

- All patients that met our diagnostic criteria for LAI or RAI.

**Exclusion criteria**:

- Patients who did not have good imaging data facilitating correct diagnosis of LAI or RAI.
- Patients who did not meet our inclusion diagnostic criteria for LAI or RAI during detailed data review process despite the fact that they were erroneously labeled or suspected as such.

From an initial number of 206 patients with the documented diagnosis CHD with heterotaxy, 16 patients were excluded so that our study cohort comprised a total number of 192 patients.

### 2.2. Variables

Each patient was evaluated for the following general variables:

- Demographic data for the patients including sex and date of birth (DOB)
- Mortality
- Duration of follow-up (calculated from date of first echo, date of birth until the date of death or date of last follow-up)

For any patient who had no documented follow-up after January 2021 until the end of our study (March 2023), we have contacted their families by phone using the documented contact information in the system to take additional information about patient’s current clinical condition and if they are still alive or not to determine mortality and survival period. Consent was received from families by phone in accordance with our IRB committed guideline, and a witness was present during the conducted phone calls. If we still were not able to contact the patients who had no follow-up documented in our system since January 2021, we considered them as missed follow-up patients.

The cases were then further evaluated for the following heart-specific variables:

**Main variables of cardio-vascular LR asymmetries**:

– Ventricular topology; D-hand or L-hand
– Orientation of the cardiac apex; left-sided, right-sided, or midline
– Aortic arch patterning; unilateral left, unilateral right, or bilateral (double aortic arch)
– Superior caval vein patterning; unilateral left, unilateral right, or bilateral
– Inferior caval vein patterning; normal or interrupted

**Associated CHDs** such as:

– Anomalies of pulmonary venous drainage; partial or total
– Atrioventricular septal defect; balanced or unbalanced
– Common atrial cavity
– Functionally univentricular heart (FUVH) including the morphological identity of its dominant ventricle
– Hypoplastic left heart syndrome
– Transposition of the great arteries
– Double outlet right ventricle
– Anatomy of pulmonary valve; stenosis, atresia, or normal
– Aortic arch abnormalities; coarctation, interruption, or hypoplasia

**Associated congenital disorders of excitation and conduction**:

– Generation of atrial activity (determined by the p-wave axis) divided into 4 groups:
  - Left inferior axis (0 to +90)
  - Left superior axis (0 to −90)
  - Right inferior axis (+90 to 180)
  - Right superior axis (−90 to 180)
– We further analyzed generation of atrial activity by combining the above 4 axis to another 4 groups
  - Left axis (combining left inferior axis and left superior axis)
  - Right axis (combining right inferior axis and right superior axis)
  - Inferior axis (combining left inferior axis and right inferior axis)
  - Superior axis (combining left superior axis and right superior axis)
– Presence of documented atrial arrhythmias (SVT, atrial fibrillation, atrial flutter, atrial tachycardia)
– Conduction abnormalities (complete heart block, junctional rhythm whether inter mittent or permanent).

***NOTE:*** Complete heart block was grouped into three categories:

- Complete heart block (congenital)
- Complete heart block (post-op)
- Complete heart block (acquired)

– Ventricular arrhythmias (ventricular fibrillation, ventricular tachycardia)
– Permanent pacemaker insertion

After the above listed variables were established, the principal investigator (T.O.) reviewed all the data from echoes, cardiac MRIs, cardiac CT, ECG, and 24 h Holter monitoring and collected and filed all the data according to the listed variables. The data was reviewed one more time with a senior cardiologist (one of the co-investigators, A.M.A.A.) to confirm that all variables are correct. After collecting all data for and from the two groups, we analyzed our results in the master data file to determine the percentages of each variable from the total number of patients and the relevance between each variable.

Finally, we analyzed our data to search for:

(1) The proportions of ventricular D- and L-hand topology in the whole study population.
(2) The proportions of ventricular D- and L-hand topology in cases of (a) LAI, and (b) RAI.
(3) Statistically significant differences in mortality between LAI and RAI patients.
(4) Statistically significant associations between the type of ventricular topology (D-hand, L-hand) and mortality (a) in the whole study population and (b, c) in the LAI and RAI subsets.
(5) Statistically significant associations between the type of ventricular topology (D-hand, L-hand) and orientation of the cardiac apex, aortic arch patterning, and patterning of SVC and IVC (a) in the whole study population and (b, c) in the two study subsets (LAI, RAI).
(6) Statistically significant associations between the type of ventricular topology (D-hand, L-hand) and specific CHDs (AVSD, functionally univentricular heart, common atrial cavity, TGA, DORV, PV defects, anomalies in pulmonary and systemic venous drainage) (a) in the whole study population and (b, c) in the two study sub-populations (LAI, RAI).
(7) Statistically significant associations between the type of ventricular topology (D-hand, L-hand) and congenital disorders of excitation and conduction (a) in the whole study population and (b, c) in the two study subsets (LAI, RAI).

### 2.3. Study Design and Data Collection

This retrospective cross-sectional study was conducted at a single center (KACC). All data supporting the findings of this study are available from the corresponding author upon reasonable request. Data were collected according to the inclusion and exclusion criteria for patients with LAI and RAI, as described in detail above.

We used the following digital programs for data collection: *IntelliSpace Cardiovascular 8* (Philips), representing our digital imaging database, *MUSE Cardiology Information System NX 10.1.8.20532* (GE HealthCare), representing our ECG and Holter digital database, and *BESTCare2.0A*, representing our electronic medical record (EMR) system. The data were extracted by our clinical pediatric research technician in the cardiac center and provided to us in an Excel spreadsheet.

For our present retrospective study, diagnosis of LAI or RAI was primary based on data from advanced imaging modalities (CT, MRI) whenever available (n = 108). Thereby, a diagnosis was based on the morphology of the lungs and airways or on the morphology of the atrial appendages as described previously [9]. Only if CT or MRI data were not available (n = 84), we used imaging data from echocardiography and abdominal US.

The morphological phenotypes of the hearts were analyzed according to the sequential segmental approach [17]. For the echocardiographic diagnosis of LAI or RAI we used the standard echocardiographic view criteria (from abdominal transverse plane of the so-called sub-xiphoid (subcostal) views), including findings that are consistent with either LAI or RAI [43,44]. Thereby, the following definitions were used: LAI or RAI [43,44]. Thereby, the following definitions were used:

(8) Usual arrangement (situs solitus) is when the aorta and inferior caval vein lie apart, on opposite sides of the spine, with the aorta on the left.
(9) Mirror-imagery (situs inversus) is the mirror-imaged arrangement with the aorta on the right and the inferior caval vein on the left.
(10) RAI is when the aorta and inferior caval vein are on the same side of the spine, with the vein slightly anterior.
(11) LAI is when the aorta is in the midline and the azygos vein is located in a posterolateral position.

Ventricular topology was determined by one of the co-investigators (A.M.A.A.), who is an expert in advanced cardiac imaging. Diagnosis was made according to the following approach (as described and discussed by [45,46]:

- Morphologic identities of the ventricles were identified based on their distinct morphological features.
- Ventricular topology was determined using the "handedness" method, independent of bodily orientation. Right-hand topology corresponds to D-looped ventricles, and left-hand topology corresponds to L-looped ventricles.

In cases where the morphological RV lacked its inlet component, ventricular topology was inferred from the positioning of the incomplete ventricle (right-anterior position of the incomplete RV is suggestive of D-hand topology, left-anterior position of the incomplete RV is suggestive of L-hand topology). The diagnostic accuracy was significantly improved through the use of 3D volume reconstructed modules generated from high-quality advanced cardiac imaging data.

Associated congenital disorders of excitation and conduction were evaluated by reviewing the ECGs of all patients, including 24 h Holter monitor data, if available. Electrophysiology consultant colleagues (mentioned in the Acknowledgement Section) were consulted for specific questions regarding the type of p-wave axis and heart block patterns.

Mortality was defined as all confirmed deaths within the study population. This was determined by aggregating all documented deaths in our database and through follow-up phone calls. Patients were considered alive if they had a documented hospital visit within the two years prior to the end of the study period (January 2021 to March 2024). For patients without documented follow-up, phone contact was attempted with family members after obtaining appropriate consent in accordance with IRB guidelines. Their status (alive or deceased) was then confirmed. Patients without documented follow-up and whose families could not be reached due to incorrect or disconnected phone numbers were categorized as having missed follow-up.

Once all the relevant patient populations and groups were identified, a data Masterfile was created in Excel (Microsoft Office, Version 2010) with all the defined variables mentioned above to answer the set of questions that we had outlined for our study.

### 2.4. Statistical Analysis

Data were presented as a mean ± standard deviation (SD) for continuous variables. Data that did not fit a normal distribution were expressed as a median and an interquartile range (IQR). Categorical variables were presented as numbers and percentages. If required for advanced data analysis, other statistical tools, such as the chi-squared test or Fisher’s exact test, were used to compare categorical data, and an independent sample *t*-test was used to compare continuous data. A difference between the compared data was regarded as significant when the *p*-value was ≤0.05. Survival curves (Kaplan–Meier curves) were created, and mortality was compared between groups using the Breslow (Generalized Wilcoxon) test. Analysis was performed using the Statistical Package for the Social Sciences (SPSS) software for Windows (version 22, IBM Corp, Armonk, NY, USA).

### 2.5. Ethical Considerations

This research was approved by the ethical research committee within the Institutional Review Board (IRB) at the center’s medical research center on 7 May 2023, and the IRB research protocol number is RC19/204/R. Consent to call patients who missed follow-up for over 2 years by phone was obtained and they were contacted about the current status of patients now (alive or not). There was no need for a consent form for the rest of the data since our data collection method consisted mainly of a chart review for the remaining variables. Patient confidentiality was maintained at all levels as only the PI and co-investigators had access to the data and were able to collect it. In addition, the data collected were kept in a secure place, using computerized methods in three specific password-protected computers, and by using coded serial numbers, we insured that the data did not contain any identification of the patients included in the research. None of the patient’s medical record numbers (MRN) were sent with the data for analysis as well.

## 3. Results

**Figure 1:**
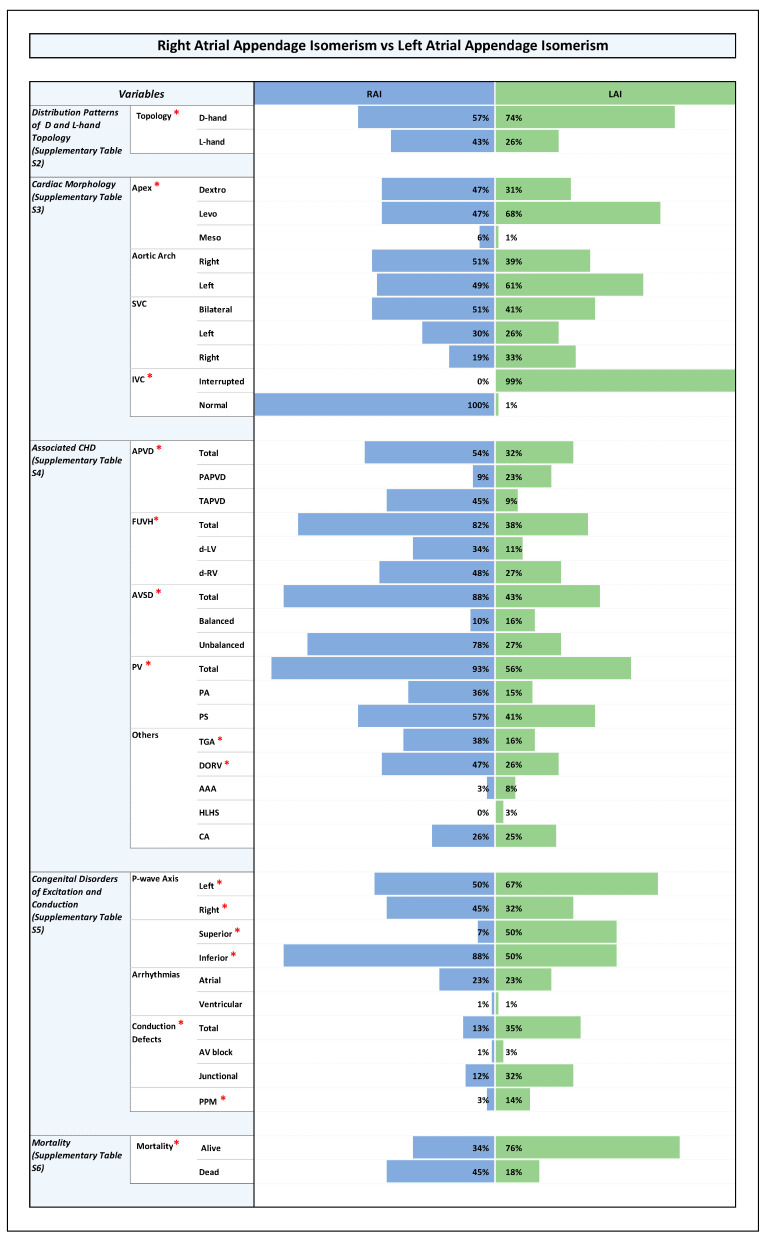
Histogram showing frequencies of variables in right atrial appendage isomerism vs. left atrial ap-pendage isomerism. Note: Statistically significant *p*-value results were marked with a red star next to the significant variable in the list of variables to the left side of the graph.

**Figure 2:**
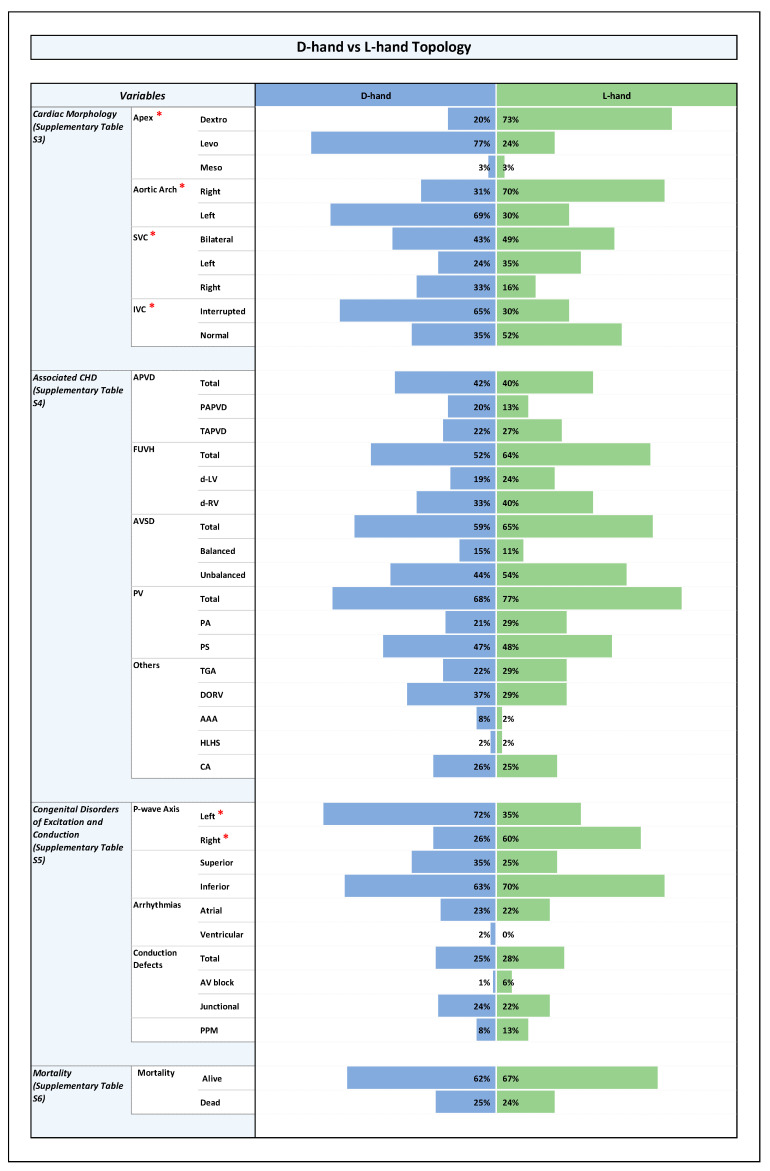
Histogram showing frequencies of variables in D-hand vs. L-hand topology. Note: Statistically significant *p*-value results were marked with a red star next to the significant variable in the list of variables to the left side of the graph.

**Figure 3:**
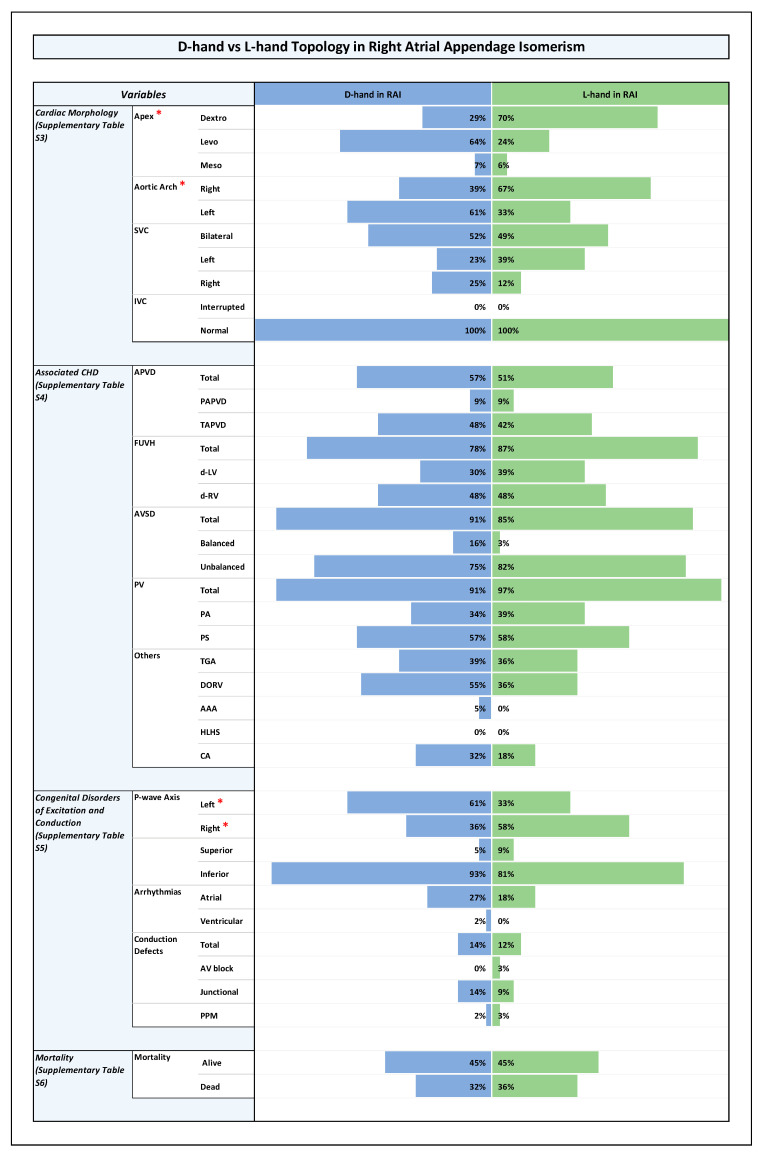
Histogram showing frequencies of variables in D-hand vs. L-hand topology in the setting of right atrial appendage isomerism. *Note:* Statistically significant *p*-value results were marked with a red star next to the significant variable in the list of variables to the left side of the graph.

**Figure 4:**
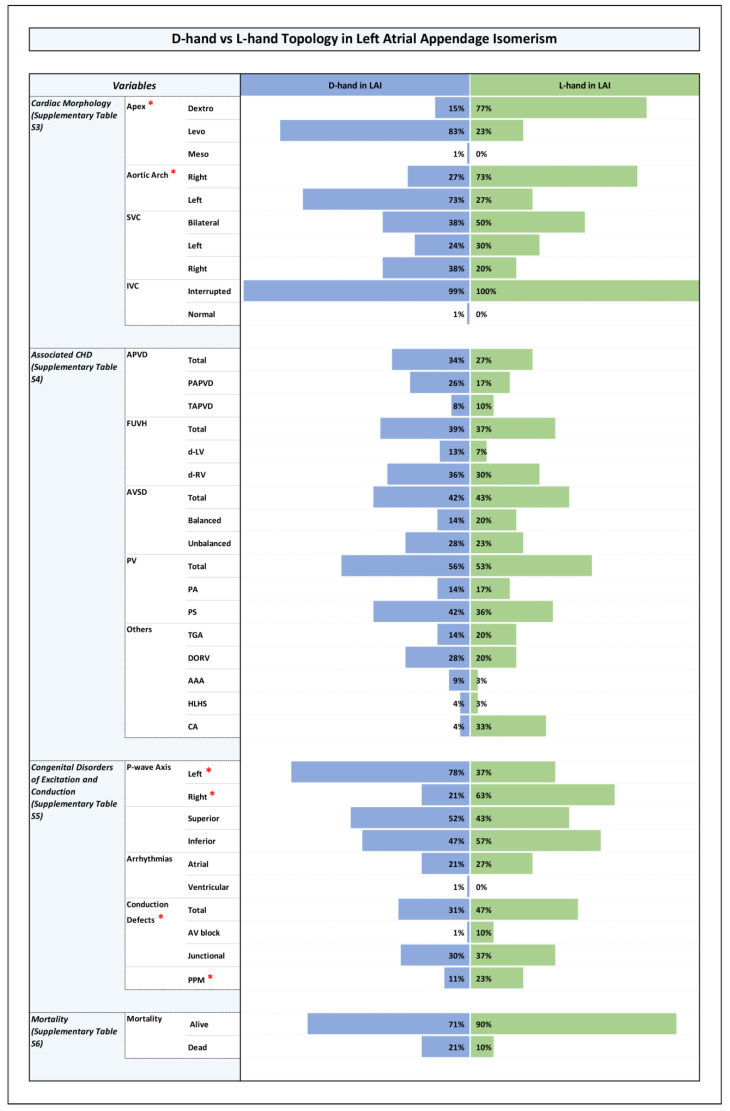
Histogram showing frequencies of variables in D-hand vs. L-hand topology in the setting of left atrial appendage isomerism. Note: Statistically significant *p*-value results were marked with a red star next to the significant variable in the list of variables to the left side of the graph.

All results are shown in detail in tables within the ‘Supplementary Materials’ Section (Supplementary Tables S1–S7). The key findings are summarized here and presented in the form of figures (Figure 1, Figure 2, Figure 3 and Figure 4).

### 3.1. Size of Study Population and Subsets (Figure 1 and Supplementary Table S2)

The whole study population comprised 192 patients. The median age at diagnosis was 1.1 months (interquartile range(IQR) 0.2–6.7 months). From these patients, 115 were assigned to the LAI subset and 77 were assigned to the RAI subset.

### 3.2. Sex Distribution

As shown in Supplementary Table S1, there was an almost equal distribution of sexes within the whole study population as well as among the LAI and RAI subsets.

### 3.3. Distribution of Ventricular Topologies (Figure 1 and Supplementary Table S2)

Among the whole study population, there was a bias toward D-hand topology (67% vs. 33%). A statistically significant bias toward the presence of ventricular D-hand topology was also found among LAI patients (74% vs. 26%). The RAI subset, in contrast, showed an almost equal distribution of D-hand and L-hand topologies (57% vs. 43%).

### 3.4. Orientation of the Cardiac Apex and Patterning of Aortic Arch, SVC, and IVC (Figure 1, Figure 2, Figure 3 and Figure 4 and Supplementary Table S3)

#### 3.4.1. Orientation of the Cardiac Apex

Among LAI patients, there was a statistically significant bias towards the normal arrangement (levo-position), while RAI patients had an almost equal distribution of left-sided and right-sided cardiac apex with only a small group having midline cardiac apex.

When we analyzed the relation between ventricular topology and the orientation of the cardiac apex in the whole study population, we found that patients with D-hand ventricles had a statistically significant higher number of left-sided cardiac apex, while the patients with L-hand ventricles had a higher number of right-sided cardiac apex. These differences were also consistent within the LAI and RAI subsets.

These results indicate that patients with ventricular D-hand topology are more likely to have a left-sided cardiac apex while patients with ventricular L-hand topology are more prone to having a right-sided cardiac apex, regardless of whether they are found to have LAI or RAI.

#### 3.4.2. Aortic Arch Patterning (Bilateral, Unilateral Right or Unilateral Left)

Among all of our patients with heterotaxy, we never found a bilaterally symmetric aortic arch patterning (double aortic arch) but always found a bilaterally asymmetric (unilateral) pattern with an almost random occurrence of left-sided and right-sided aortic arches.

The RAI subset had an almost equal number of patients with right- and left-sided aortic arches, while the LAI subset had more patients with a left-sided aortic arch. However, this difference between the two sub-groups was not statistically significant.

When we analyzed the relation between ventricular topology and aortic arch sidedness in the whole study population, we found that patients with D-hand ventricles had a significantly higher number of left-sided aortic arches, while patients with L-hand ventricles had a higher number of right-sided aortic arches. These differences were also consistent within the LAI and RAI subsets.

These results indicate that patients with ventricular D-hand topology are more likely to have a left-sided aortic arch, while patients with L-hand ventricles are more prone to have a right-sided aortic arch, regardless of if they are found to have LAI or RAI.

#### 3.4.3. Patterning of SVC (Bilateral, Unilateral Right or Unilateral Left)

Among all of our patients with heterotaxy, a bilaterally symmetric SVC patterning was found in almost half of the patients, while a bilaterally asymmetric (unilateral) pattern was found in the other half with random occurrence of left-sided and right-sided SVC.

Principally almost the same situation was found in the LAI as well as RAI subsets.

When we analyzed the relation between ventricular topology and SVC patterning in general, we found that, among the whole study group, there was no statistically significant association between the type of ventricular topology and the occurrence of a bilateral or a unilateral pattern. The same holds true for the LAI and RAI subsets.

However, when we analyzed the relation between ventricular topology and the position of a unilateral SVC, we found that there was a statistically significant association between the type of ventricular topology and the sidedness of the vein. Among patients with D-hand ventricles, there were more cases of a unilateral right SVC, and patients with L-hand ventricles had more cases of a unilateral left SVC.

#### 3.4.4. Patterning of IVC (Normal or Interrupted)

Interruption of IVC is generally recognized as a good indicator for LAI. It was found, however, also in patients with a normally structured heart [47] and even in cases of RAI [48,49]. Our diagnosis of LAI was based on the ultrasonographically detected presence of an interrupted IVC and azygos continuation in a relatively large number of cases for which CT or MRI data was not available (see M and M). It was therefore expected that only a minority of our patients with RAI might display an interrupted IVC, whereas the vast majority of our patients with LAI were expected to display interrupted IVC. We indeed found that all of our patients with RAI had normal IVC, whereas almost all patients with LAI had an interrupted IVC.

Among the whole study population, patients with a D-hand ventricle had a higher incidence of interrupted IVC compared to cases with normal IVC, whereas there was an almost equal distribution of interrupted and normal IVC among patients with an L-hand ventricle. As all RAI patients were found to have normal IVC, no useful statistical analysis could be performed to compare patients with D- and L-hand ventricles within this group. In LAI patients, no statistically significant difference was noticed between patients with D- and L-hand ventricles as all patients with LAI, except for one, had interrupted IVC.

### 3.5. Associated CHDs (Figure 1, Figure 2, Figure 3 and Figure 4 and Supplementary Table S4)

Among our patients the following CHDs were found:

– Aortic arch abnormalities (coarctation, interruption, or hypoplasia)
– Anomalies in pulmonary venous drainage (partially anomalous, totally anomalous)
– Atrioventricular septal defect (balanced or unbalanced)
– Functionally univentricular hearts with a dominant ventricle of left or right morphology
– Hypoplastic left heart syndrome
– Common atrial cavity
– Transposition of the great arteries
– Double outlet right ventricle
– Anomalies of pulmonary valve (pulmonary stenosis, pulmonary atresia)

When we analyzed the distribution pattern of the above-listed CHDs among our study subsets, we found that, compared to LAI, patients with RAI had a significantly higher rate of:

1. Abnormal pulmonary venous drainage (PAPVD), mainly TAPVD.
  *Please note that in the setting of RAI, by definition, the pulmonary veins are connected anomalously, since even if returning to the heart, they are returning to an atrial chamber with a morphologically right atrial appendage.*
2. AVSD, mainly unbalanced AVSD.
3. Functionally univentricular heart, mostly with a dominant ventricle of right morphology.
  *Please note that in our patient populations data, we have not encountered any case of what some people erroneously call “single ventricle of indeterminate morphology”.*
4. Pulmonary stenosis or atresia.
5. TGA and DORV.

There were no statistically significant differences between LAI and RAI subsets with regard to the incidence of aortic arch abnormalities, hypoplastic left heart syndrome or common atrial cavity. However, the total number of patients with aortic arch abnormalities (n = 11) and hypoplastic left heart syndrome (n = 4) was too small to make a good statistical analysis.

When we analyzed the relation between ventricular topology and CHDs, we did not find significant associations between the type of ventricular topology (D-hand, L-hand) and any of the above-mentioned CHDs. There were also no statistically significant associations between the type of ventricular topology (D-hand, L-hand) and any of these heart defects within the LAI and RAI subsets.

### 3.6. Congenital Disorders of Excitation and Conduction (Figure 1, Figure 2, Figure 3 and Figure 4 and Supplementary Table S5)

With regard to inferior and superior p-wave axis, it was noted that among RAI patients there was a statistically significant higher rate of inferior p-wave axis compared to LAI patients, reflecting higher prevalence of sinus node absence or dysfunction, and higher prevalence of ectopic atrial pacemakers in patients with LAI.

Combined with the data on ventricular topology, we did not find a statistically significant difference in the presence of inferior p-wave axis between RAI hearts with D-hand and L-hand ventricles. The same holds true for patients with LAI.

With regard to left and right p-wave axis, we noted that among RAI patients there was an almost equal distribution of left and right p-wave axis. However, in the LAI group, left p-wave axis was predominant with a statistically significant higher rate, and associated with a higher prevalence of a left-sided cardiac apex in patients with LAI compared to patients with RAI.

RAI hearts with ventricular D-hand topology showed a statistically significant higher rate of left axis compared to right, reflecting the higher tendency for a left-sided cardiac apex in this particular group (see above). RAI hearts with ventricular L-hand topology showed higher rates of right axis, reflecting in turn the high rates of a right-sided cardiac apex in this setting.

LAI hearts with ventricular L-hand topology showed a statistically higher rate of right axis compared to left axis, while LAI with ventricular D-hand topology showed a higher prevalence of left axis with a statistically significant difference. The data correlates with high prevalence of a right-sided cardiac apex in L-hand group and a left-sided cardiac apex in the D-hand group.

There were no statistically significant differences in prevalence of atrial arrhythmias in all groups:

(1) RAI vs. LAI;
(2) Ventricular D-hand topology vs. L-hand topology;
(3) Ventricular D-hand topology in RAI vs. L-hand topology in RAI;
(4) Ventricular D-hand topology in LAI vs. L-hand topology in LAI.

When comparing total conduction defects—including both atrioventricular block and junctional abnormalities—a statistically significant difference was observed exclusively between the RAI and LAI subsets. The LAI group exhibited a higher incidence of conduction defects relative to the RAI group. Notably, 32% of conduction defects in the LAI group were attributable to sinus node dysfunction, compared to only 12% in the RAI group.

Looping pattern topology (D-loop vs. L-loop) showed no association with conduction defects, as the incidence was comparable between the two configurations. This lack of correlation persisted when analyzing looping patterns within the RAI and LAI subsets, with no statistically significant differences identified across all comparisons.

### 3.7. Mortality and Long-Term Survival

With regard to mortality, our data showed a statistically significant difference between the RAI and LAI subsets. In the RAI subset, more than third of the patients died, while in the LAI subset only a fifth of the patients died (Figure 1 and Supplementary Table S6).

When we analyzed the relation between ventricular topology and mortality, the following findings were made:

Among the whole study population, there was no statistically significant difference in mortality between the patients with ventricular D-hand and L-hand topology (Figure 2 and Supplementary Table S6).

The same holds true for the RAI subset (Figure 3 and Supplementary Table S6).

Among the LAI subset, however, there was a lower mortality in patients with ventricular L-hand topology compared to the patients with D-hand topology, however this difference was not statistically significant (Figure 4 and Supplementary Table S6).

**Figure 5:**
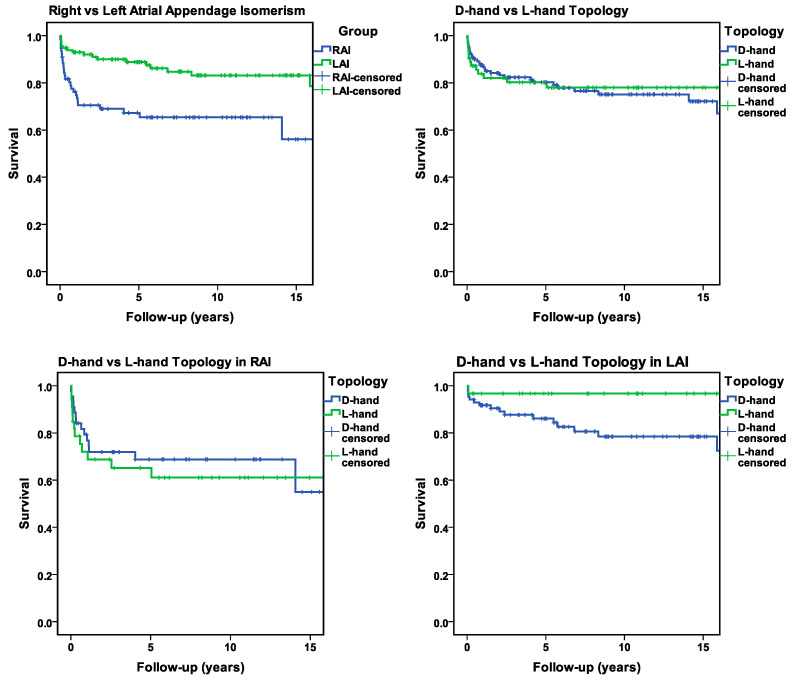
Kaplan–Meier survival curves illustrating the differences in mortality among different groups over a 15 year follow-up period. Censoring events are marked with crosses on the respective curves.

With regard to long-term survival (follow-up of at 5, 10, and 15 years) we found that RAI patients had a significantly worse outcome over the years compared to LAI patients in the same period (Figure 5 and Supplementary Table S7).

When we analyzed the relation between ventricular topology and long-term survival, the following findings were made:

Among the whole study population, there was no statistically significant difference in long-term survival between the patients with ventricular D-hand and L-hand topology (Supplementary Table S7).

In the LAI subset, however, there was a clear difference in long-term survival between the patients with D-hand and L-hand ventricles, however this difference was not statistically significant. Patients with ventricular L-hand topology showed a better long-term survival than patients with ventricular D-hand topology (Supplementary Table S7).

The same holds true for the RAI subset (Supplementary Table S7).

## 4. Discussion

The main goals of the present study were (1) to document the statistical distribution patterns of ventricular D-hand and L-hand topology in a relatively large cohort of human patients with CHDs in the setting of heterotaxy and (2) to check our patients data for the presence of significant associations between the type of ventricular topology and cardiovascular disorders as well as the patient’s survival. This was primarily performed to clarify whether the statistical distribution patterns of ventricular D-hand and L-hand topology may reflect the heterotaxy syndrome-specific tendency for visceral symmetry as predicted by two different mechanistic concepts of ventricular looping.

### 4.1. Statistical Distribution Patterns of Ventricular D-Hand and L-Hand Topology

**Figure 6:**
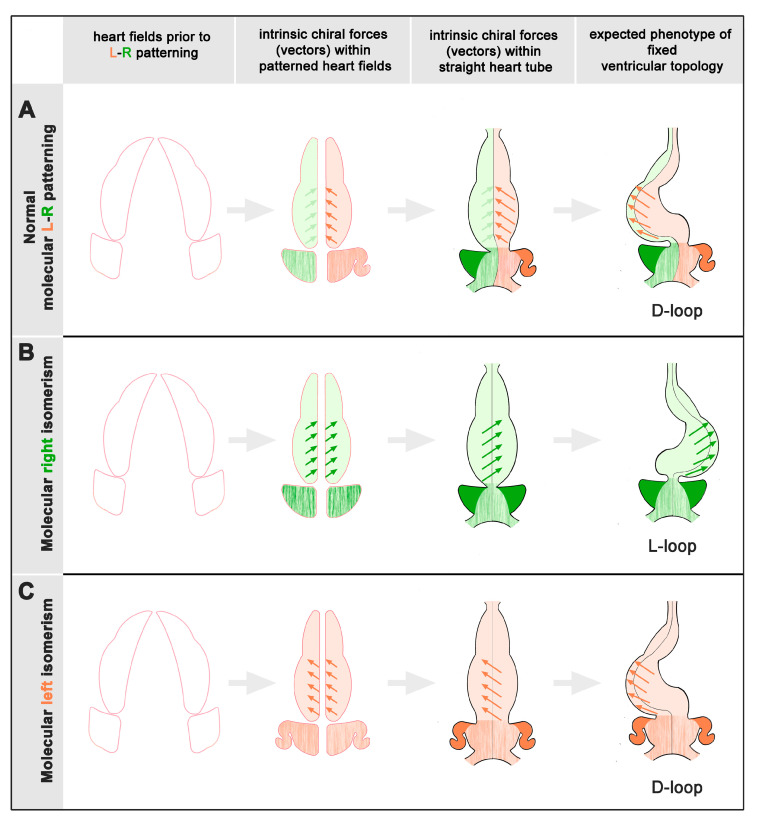
This scheme depicts scenarios of ventricular looping that are based on the concept that this process is driven by intrinsic chiral forces whose spin (D-handed (red vectors) or L-handed (green vectors)) is defined by molecular L-R patterning of the embryonic heart. Drawings show scenarios in which D-hand topology originates from intrinsic chirality within the left-sided heart fields. (**A**): Normal development. Ventricular looping is dominated by D-handed forces within the left half of the heart tube. In consequence, ventricular D-hand topology is expected to occur in almost 100% of cases. (**B**): RAI. Molecular right isomerism induces L-handed forces in both halves of the heart tube. In consequence, ventricular L-hand topology is expected to occur in almost 100% of cases. (**C**): LAI. Molecular left isomerism induces D-handed forces in both halves of the heart tube. In consequence, ventricular D-hand topology is expected to occur in almost 100% of cases.

Among the whole cohort of patients, there was a statistically asymmetric distribution pattern characterized by a bias towards D-hand topology (67% vs. 33%). When we segregated our data with regard to the two subsets of the syndrome, we found that D-hand as well as L-hand topology occurred in both subsets. There was, however, a striking difference between RAI and LAI. While the LAI patients showed a statistically significant bias toward the presence of ventricular D-hand topology (74% vs. 26%), the RAI subset showed an almost equal distribution of D-hand and L-hand topologies (57% vs. 43%). These patterns do not fit into the currently popular idea that ventricular looping is mainly driven by chiral forces intrinsic to the ventricular segment of the embryonic heart. If we assume that ventricular chirality is determined by the molecular L-R patterning of the embryonic heart, we should expect that among patients with CHDs with heterotaxy there is a binary distribution pattern in which ventricular L-hand topology should tend to occur only in one subset while D-hand topology should tend to occur only in the other subset (Figure 6).

Our data also does not seem to fit completely with the alternative hypothesis, which says that the lateral twist of the bending embryonic heart tube mainly evolves as a consequence of mechanical loads from the pericardial wall, which constrains ventral bending of the growing heart tube. According to this hypothesis, asymmetric expression of molecular L-R determinants are not responsible for the chiral deformation of the ventricular heart segment but only for generating a bias toward development of one of the two alternative phenotypes (ventricular D-hand or L-hand topology). The bias-inducing mechanism is attributed to morphological and/or functional asymmetries located at the venous and arterial poles of the linear heart tube [37,38,39,40,41], and data from mouse mutants have shown that a transient asymmetric expression of nodal at the poles of the pre-looping heart is required to amplify and coordinate these subtle asymmetries [42]. In this scenario, both the RAI as well as LAI subsets are expected to suffer from a lack of ventricular topology bias during the embryonic period of cardiogenesis and, therefore, should display a tendency for statistically symmetric distribution of ventricular D-hand and L-hand topologies; a pattern that may be regarded as a reflection of the syndrome-specific tendency for visceral symmetry at the level of the ventricular heart segment. In our patients such a tendency for symmetry was present only in the RAI subset (57% vs. 43%) while the LAI subset displayed a statistically significant bias towards D-hand topology (74% vs. 26%). In view of the ambiguous situation two questions arise: (1), does our present data correspond to previously published data and (2), how can we explain the observed deviation from a statistically symmetric distribution pattern?

Information on ventricular topology is only provided in a limited number of studies on patients with CHDs in the setting of heterotaxy. Table 1 presents the distribution patterns of ventricular D-hand and L-hand topology found in these studies. At first sight, an analysis of these data does not seem to disclose a common pattern. Some studies present statistically almost-symmetric distributions of ventricular D-hand and L-hand topologies among RAI as well as LAI patients [17,50,51]. Others present distribution patterns similar to our data [12], and the largest group of studies presents asymmetric distribution patterns that are found in RAI as well as LAI and are characterized by a moderate (~60:40) bias [48,49,52,53,54]. Interestingly, this bias was towards the presence of D-hand topology in all but a single study [55]. Pooling of the data from all studies shown in Table 1 discloses that the bias toward ventricular D-hand topology is stronger in LAI (63.8%) than RAI (55.4%). This finding principally corresponds to our data although we observed a much stronger bias toward D-hand topology among our LAI patients (74% vs. 26%).

How can we explain the above-described deviations from a statistically symmetric distribution? One possible explanation could be a higher rate of prenatal death among heterotaxy patients with ventricular L-hand topology as compared to patients with ventricular D-hand topology, especially in the subset of LAI. This idea is suggested by the following facts: (1) The prenatal mortality of fetuses with CHDs with heterotaxy is high in the presence of hydrops caused by congenital atrioventricular block [57,58]. (2) There is evidence for a strong association between the presence of ventricular L-hand topology and anomalies in atrioventricular conduction axis among the RAI as well as LAI subsets [15]. (3) Congenital heart block preferentially affects LAI patients [58].

Our present data from postnatal patients did not uncover any statistically significant associations between ventricular topology and the presence of life-threatening congenital arrhythmias or complex CHDs. Moreover, we made the unexpected and somewhat puzzling finding that, among our LAI patients, the rate of long-term survival was higher in patients with ventricular L-hand topology compared to those with D-hand topology (Figure 5). We have no sound explanation for this finding. It is unclear as to whether this finding reflects the natural prognosis of the patients or results from therapeutic interventions. Clarifying this question, and identifying the underlying factors, may be of clinical importance but would go beyond the scope of the present paper. Thus, future studies are needed to clarify the situation.

A second cause for the above-described deviations from statistically symmetric distributions might be seen in a heart segment-specific patterning of molecular L-R identities. Campione et al. [23] have shown that mouse embryos with heterotaxy can display molecular isomerism not only in the heart-forming fields but also in the atrial and ventricular segments of embryonic hearts. A corresponding observation was made in chick embryos [59]. Campione et al. have furthermore shown that molecular isomerism of the atrial and ventricular segments did not always occur together. The reasons for this phenomenon are unknown, but its existence suggests that the molecular L-R identities of the heart are modulated by heart segment-specific signaling pathways [23]. Such activation pathways might be responsible for the slight to moderate dominance of D-hand topology in the RAI and LAI subsets of human heterotaxy patients.

A third cause might be attributed to the composition of study populations. Data from animal models and human patients have shown that the clinical diagnosis *heterotaxy* characterizes a genetically very heterogeneous entity [60]. Studies on animal models, furthermore, have shown that, with regard to ventricular topology, we principally can distinguish between two different types of genetic animal models for heterotaxy: (1) those in which the presence of atrial isomerism is regularly associated with randomization of ventricular topology and (2) those in which atrial isomerism is associated with ventricular D-hand topology, only. While the first type encompasses the vast majority of animal models for heterotaxy (e.g., [61,62,63]), the second type encompasses only a relatively small group, which includes mutations/defects in lefty1 [64], sonic hedgehog [65,66], and Pitx2 [67]. The existence of animal models for heterotaxy with normal bias towards ventricular D-hand topology suggests that the statistical distribution pattern of ventricular D-hand and L-hand topology found among cohorts of human patients with heterotaxy might depend on the proportion of patients carrying mutations in one of the three above-mentioned genes. At the present time, it is unknown why these mutants retain the normal bias towards development of ventricular D-hand topology. Based on the above-mentioned data from Campione et al., however, we can speculate that this might be related to a heart segment-specific modulation of molecular L-R identities.

### 4.2. Cardiovascular Anomalies in the Setting of Heterotaxy, Their Distribution Among RAI and LAI Subsets, and Associations with Ventricular D-Hand and L-Hand Topology

It is well known that heterotaxy is frequently associated with CHDs and that the two subsets of the syndrome (RAI, LAI) differ from each other with regard to the pattern of associated cardiovascular disorders and with regard to clinical prognosis [7,8,54]. The patterns found in the present study correspond to these well-known patterns (Figure 1). When we looked for statistically significant associations between the type of ventricular topology and cardiovascular disorders, we found that, among the whole cohort as well as among the RAI and LAI subsets, there were no significant differences between the two variants of ventricular topology with regard to the prevalence of cardiovascular disorders that have a strong influence on the prognosis of patients with heterotaxy (Figure 2, Figure 3 and Figure 4). There were a few anatomical features of minor prognostic importance, however, that displayed statistically significant associations. These features are as follows: (1) position of the cardiac apex; (2) direction of the p-wave axis; and (3) aortic arch sidedness (Figure 2, Figure 3 and Figure 4). In the following paragraphs, these three features will be discussed with regard to the embryology of the situs of the cardiovascular system.

**Position of the cardiac apex**. In articles on the embryology of visceral asymmetries, the normally left-sided position (levo-position) of the apex of the human heart is frequently described as a feature that reflects the looping morphogenesis of the embryonic heart. According to this view, we should expect that, in the setting of heterotaxy, ventricular D-hand topology should be regularly associated with the levo-position of the cardiac apex, while ventricular L-hand topology should display a regular association with the dextro-position of the cardiac apex. Our present data disclosed statistically significant associations between ventricular D-hand topology and levo-position of the apex (64–83%), and ventricular L-hand topology and dextro-position (70–77%). We should emphasize, however, that our data, as well as previous data (see Figure 5 in [19]), do not indicate a regular association. We therefore have to conclude that the direction of embryonic heart looping seems to play a strong but not exclusive role in positioning of the cardiac apex.

**Direction of p-wave axis.** With regard to this feature, we found that D-hand topology was associated with the presence of left p-wave axis (61–78%), while L-hand topology was associated with the presence of right p-wave axis (58–63%). It is well known that the direction of p-wave axis corresponds to the general position of the cardiac apex [68,69]. Our findings of a higher percentage of levo-position of cardiac apex in cases with D-hand topology and a higher percentage of dextro-position in cases with L-hand topology may reflect the topological influence on the apex position which corresponds to the general direction of the p-wave axis. In regard to the vertical orientation of the p-wave axis, we observed a significantly higher prevalence of inferior p-wave axis among RAI patients (88%) compared to LAI patients (50%), suggesting that sinus node dysfunction and ectopic atrial pacemakers may be more frequent in the LAI group.

**Aortic arch sidedness**. The normal arrangement of the human aortic arch and its major branches is characterized mainly by the presence of a single, left-sided aortic arch and represents a well-known example of visceral asymmetry near to the center of our cardiovascular system. It results from asymmetric remodeling of the originally bilateral symmetric pharyngeal arch arterial system during the embryonic period of prenatal development [70,71,72]. It is tempting to speculate that, in the setting of heterotaxy, the aortic arch system should display a tendency for bilateral symmetry, characterized by a high prevalence of double aortic arch. Surprisingly, however, such arrangements do not characterize the heterotaxy syndrome of humans [10] as well as animal models [73]. Previous studies, as well as our present data, show that a right aortic arch is a very frequent finding among patients with heterotaxy [74,75,76]. With regard to statistical distribution patterns of left and right aortic arches, we found that the whole cohort of our patients, as well as the two subsets of the syndrome, all displayed almost symmetric distribution patterns, that showed only small or moderate but statistically non-significant bias towards the occurrence of a left aortic arch (whole cohort: 56.25% vs. 43.75%; RAI: 49% vs. 51%; LAI: 61% vs. 39%). This finding seems to correspond to a previous statement that heterotaxy is characterized by randomization of aortic arch sidedness [73]. We should note, however, that our data does not correspond to all previously published data. As shown in Table 2, there is a great variability among the data from previous studies, which may be explained in part by low case numbers. Pooling of these data discloses distribution patterns that are closer to randomization but show a small (RAI) to moderate (LAI) bias toward the presence of a left aortic arch.

It appears that, in the setting of heterotaxy, the sidedness of the aortic arch seems to represent a binary anatomical variable that can exist either in the form of a right-sided or left-sided aortic arch. A bilaterally symmetric third form (double aortic arch) virtually does not occur in the syndrome. Thus, the syndrome-specific tendency for visceral symmetry usually does not appear in the aortic arch system of individual patients. The present data as well as previously published data, however, suggest that the syndrome-specific tendency for visceral symmetry becomes apparent at the population level where it appears as a tendency for the statistically symmetric (randomized) distribution of right- and left-sided aortic arches. This situation resembles the above-described situation found at the ventricular heart segment of patients with CHDs with heterotaxy. In the setting of heterotaxy, ventricular topology and aortic arch sidedness have previously been described as randomized features. In view of this fact, it seems to be a mystery that our study is the first to check patients’ data for the presence of statistically significant links between the two anatomical variables. Our data show that, in the setting of heterotaxy, there is a statistically significant association between ventricular D-hand topology and the presence of a left aortic arch (whole cohort: 69%; RAI: 61%; LAI: 73%), while ventricular L-hand topology is associated with a right aortic arch (whole cohort: 70%; RAI: 67%; LAI: 73%).

Do these associations reflect ontogenetic relationships? In the setting of heterotaxy, the emergence of a unilateral aortic arch can hardly be ascribed to molecular determinants of body sidedness since these determinants should be expressed in bilaterally symmetric fashions. It is therefore tempting to speculate that asymmetric remodeling of the embryonic pharyngeal arch arterial system is mainly controlled by other factors such as hemodynamic load. Data from animal models for heterotaxy and flow simulation studies, indeed, support the hypothesis that asymmetric remodeling of the pharyngeal arch arterial system is mainly driven by asymmetric routing of blood flow in consequence of chiral morphogenesis of the ventricular outflow tract [73,77]. We therefore think that the associations between ventricular topology and aortic arch sidedness, found in the present study, reflect the dependence of pharyngeal arch arterial remodeling on ventricular looping.

## 5. Limitations

Due to the rarity of heterotaxy syndrome, our present study comprised only a limited number of patients (n = 192), even though we looked through data that spans around 22 years.

Although our diagnosis of heterotaxy and its subsets was based on MRI and CT image data in the majority of our patients (n = 108), there was a relatively large number of cases (n = 84) in which the diagnosis was based on echo data only. We are aware of the fact that, due to this limitation, a small but unknown number of cases, e.g., LAI without interrupted IVC, may have received a wrong subset diagnosis.

With regard to survival and mortality data, we had a few patients (n = 23) who lacked a completely documented follow-up and could not be reached by phone (wrong or disconnected phone number).

With regard to rhythm abnormalities, we should note that some patients might have undocumented arrhythmias that were not seen in Holter monitoring or documented during their admission periods. Some patients also had very limited numbers of ECGs documented in the system either because they are still infants, or because they died during infancy without having Holter monitoring or multiple ECGs performed for them to see their rhythm problems in the long term. Further, around five patients had no documented ECGs in the system, and some patients died in early infancy, which made any judgment regarding late presentations of heart blocks or junctional rhythm impossible. Thus, this also affected the evaluation of the number of patients who needed PPM insertion.

## 6. Conclusions

Heterotaxy syndrome is characterized by a tendency for bilaterally symmetric arrangement of the inner organs. In the heart and aortic arch system of an affected patient, this tendency is regularly apparent only at the level of the atrial segment. The situs of the ventricular heart segment and the anatomical pattern of the aortic arch system usually display asymmetric arrangements. In individual patients, the ventricular segment can show either D-hand or L-hand topology, and the aortic arch is either left- or right-sided. It appears that, in the setting of heterotaxy, ventricular topology and aortic arch sidedness seem to behave as binary anatomical variables. Among large cohorts of patients with CHDs in the setting of heterotaxy, the statistical distribution patterns of both of these binary variables show a tendency for statistically symmetric distribution, although there is a slight to moderate bias toward the presence of a D-hand topology and left aortic arch. The bias may be explained by a higher rate of fetal death among patients with ventricular L-hand topology as compared to patients with D-hand topology, possibly caused by congenital heart block. Our present data from postnatal patients, however, did not uncover a statistically significant association of L-hand topology with congenital atrioventricular conduction defects. The tendency for statistically symmetric distribution patterns of ventricular topology and aortic arch sidedness among patients with heterotaxy is interpreted as reflecting the syndrome-specific tendency for bilateral symmetry at the population level.

## Figures and Tables

**Table 1:** Ventricular topology in patients with cardiac malformations in the setting of heterotaxy (data from previous studies).

	RAI				LAI			
Study	Case No.	D-Hand	L-Hand	Undetermined	Case No.	D-Hand	**L-Hand**	**Undetermined**
Stanger et al. [55]	23 *	34.8% (8)	65.2% (15)		17 *	70.6% (12)	29.4% (5)	
Carvalho et al. [50]	13	54% (7)	46% (6)		12	50% (6)	50% (6)	
Vairo et al. [51]					28	53% (15)	47% (13)	
Francalanci et al. [52]	33 *	60.6% (20)	39.4% (13)					
Ho et al. [53]	10 *	60% (6)	40% (4)		20 *	65% (13)	10% (2)	25% (5)
Uemura et al. [12]	125 *	54% (68)	42% (52)	4% (5)	58 *	79% (46)	16% (9)	5% (3)
Smith et al. [15]	10 *	20% (2)	20% (2)	60% (6)	25 *	60% (15)	32% (8)	8% (2)
Yildirim et al. [56]	43	53.5% (23)	18.6% (8)	27.9% (12)	88	59.1% (52)	22.7% (20)	18.2% (16)
Loomba et al. [17]	37 *	50%	42%	8%	12 *	47%	53%	
Tremblay et al. [54]	131 *	61% (80)	36% (47)	3% (4)	56 *	66% (37)	34% (19)	
Kiram et al. [48]	184	57.6% (106)	41.9% (77)	0.5% (1)	118	66.1% (78)	33.1% (39)	0.8% (1)
Oreto et al. [49]	43	60.5% (26)	39.5% (17)		35	57% (20)	43% (15)	
Pooled data	652	55.4% (361)	39.9% (260)	4.7% (31)	469	63.8% (299)	30.5% (143)	5.7% (27)

* Postmortem analysis. Green background color highlights the subset of RAI, and purple color highlights the subset of LAI. Olive green background marks the pooled data.

**Table 2:** Aortic arch sidedness in patients with cardiac malformations in the setting of heterotaxy (data from previous studies).

	RAI					LAI				
Study	Case No.	Left Arch	Right Arch	Double Arch	Undetermined	Case No.	Left Arch	Right Arch	Double Arch	Undetermined
Ho et al. [53]	10 *	50% (5)	50% (5)			20 *	75% (15)	25% (5)		
Francalanci et al. [52]	33 *	60.6% (20)	39.4% (13)							
Smith et al. [15]	10 *	90% (9)	10% (1)			25 *	88% (22)	12% (3)		
Loomba et al. [17]	37 *	70% (26)	30% (11)			12 *	95% (11)	5% (1)		
Tremblay et al. [54]	131 *	63% (77)	37% (46)		8	57 *	75% (41)	25% (15)		1
Kiran et al. [48]	184	46.7% (86)	53.3% (98)			118	60% (71)	40% (47)		
Oreto et al. [49]	43	70% (30)	30% (13)			35	51% (18)	49% (17)		
Pooled data	448	56.5% (253)	41.7% (187)		1.8% (8)	267	66.6% (178)	33% (88)		0.4% (1)

* Postmortem analysis. Green background color highlights the subset of RAI, and purple color highlights the subset of LAI. Olive green background marks the pooled data.

## Data Availability

The original contributions presented in this study are included in the article/Supplementary Material. Further inquiries can be directed to the corresponding author.

## Supplementary Materials

The following supporting information can be downloaded at: https://www.mdpi.com/article/10.3390/jcdd12110430/s1, Table S1: Distribution of sexes in patients with right versus left atrial appendage isomerism and ventricular D-hand and L-hand topology, respectively; Table S2: Distribution patterns of ventricular D-hand and L-hand topology among patients with left and right atrial appendage isomerism; Table S3: Position of the cardiac apex and patterning of aortic arch, SVC, and IVC in different groups; Table S4: Associated congenital heart diseases in different groups; Table S5: Congenital disorders of excitation and conduction in different groups; Table S6: Mortality in different groups; Table S7: Survival at 5 years, 10 years and 15 years, respectively in different groups.

## Abbreviations

AAA	Aortic arch abnormalities
APVD	Anomalous pulmonary venous drainage
AV	Atrioventricular
AVSD	Atrioventricular septal defect
CA	Common atrial cavity
CHB-c	Complete heart block (congenital)
CHB-ac	Complete heart block (acquired)
CHB-po	Complete heart block (post-op)
CHD	Congenital heart disease/defects
Dextro	Dextro-position of cardiac apex
D-hand	D-hand topology
d-LV	Dominant left ventricle
DORV	Double outlet right ventricle
d-RV	Dominant right ventricle
ECG	Electrocardiogram
FUV	Functionally univentricular heart
HLHS	Hypoplastic left heart syndrome
IVC	Inferior vena cava
IRB	Institutional Review Board
JIR	Junctional intermittent rhythm
JPR	Junctional persistent rhythm
KACC	King Abdulaziz Cardiac Center
KAMC	King Abdulaziz Medical City
LAI	Left atrial appendage isomerism
Levo	Levo-position of cardiac apex
LIA	Left inferior axis (0 to +90)
LSA	Left superior axis (0 to −90)
L-hand	L-hand topology
LR	Left right
Meso	Meso-position of cardiac apex
MRN	Medical record number
ODS	Online data supplements
P	*p*-value
PA	Pulmonary atresia
PAPVD	Partially anomalous pulmonary venous drainage
PPM	Permanent pacemaker
PS	Pulmonary stenosis
PV	Pulmonary valve
RAI	Right atrial appendage isomerism
RIA	Right inferior axis (+90 to 180)
RSA	Right superior axis (−90 to 180)
SA	Sinoatrial
SVC	Superior vena cava
TAPVD	Totally anomalous pulmonary venous drainage
TGA	Transposition of the great arteries
V	Ventricle
