# Supplementary material for: Ventricular Topology in Congenital Heart Defects Associated with Heterotaxy: Can We Find Patterns Reflecting the Syndrome-Specific Tendency for Visceral Symmetry?
Source: J Cardiovasc Dev Dis. 2025 Oct 31;12(11):430. doi: 10.3390/jcdd12110430 (PMC12653055; doi:10.3390/jcdd12110430)
Supplement: Supplementary file 1 [file jcdd-12-00430-s001.zip › jcdd-3862430-supplementary.pdf]

Supplementary Materials

Supplementary Tables S1-S7

Table S1:

Distribution of sexes in patients with right versus left atrial appendage isomerism and ventricular D-hand and L-hand topology, respectively

Sex	Groups n= 192	RAI n=77	vs	LAI n=115	P	D-hand n=129	vs	L-hand n=63	P
Male	95 (49.5%)	43 (56 %)		52 (45 %)	0.149	59 (46 %)		36 (57%)	0.138
Female	97 (50.5%)	34 (44%)		63 (55%)		70 (54%)		27 (43%)	

Table S2:

Distribution patterns of ventricular D-hand and L-hand topology among patients with left and right atrial appendage isomerism

Sub-Groups	D -hand	vs	L- hand	P
RAI (n=77)	44 (57%)		33 (43%)	0.015
LAI (n=115)	85 (74%)		30 (26%)	
Total (n =192)	129 (67 %)		63 (33%)	

Table S3:

Position of the cardiac apex and patterning of aortic arch, SVC, and IVC in different groups

Groups n= 192	RAI n=77 (%)	vs	LAI n=115(%)	P	D-hand n=129(%)	vs	L-hand n=63(%)	P	D-hand in RAI n=44(%)	vs	L-hand in RAI n=33(%)	P	D-hand in LAI n=85(%)	vs	L-hand in LAI n=30(%)	P
**Apex**																
Dextro	36 (47%)		36 (31%)	0.04	26 (20%)		46 (73%)	0.0001	13 (29%)		23 (70%)	0.002	13 (15%)		23 (77%)	0.0001
Levo	36 (47%)		78 (68%)		99 (77%)		15 (24%)		28 (64%)		8 (24%)		71 (83%)		7 (23%)	
Meso	5 (6%)		1 (1%)		4 (3%)		2 (3%)		3 (7%)		2 (6%)		1 (1%)		0 (0%)	
**Arch**																
Right	39 (51%)		45 (39%)	0.115	40 (31%)		44(70%)	0.0001	17 (39%)		22 (67%)	0.015	23 (27%)		22 (73%)	0.0001
Left	38 (49%)		70 (61%)		89 (69%)		19 (30%)		27 (61%)		11 (33%)		62 (73%)		8 (27%)	
**SVC**																
Bilateral	39 (51%)		47 (41%)	0.12	55 (43)%		31 (49%)	0.032	23 (52%)		16 (49%)	0.18	32 (38%)		15 (50%)	0.21
Left	23 (30%)		30 (26%)		31 (24%)		22 (35%)		10 (23%)		13 (39%)		21 (24%)		9 (30%)	
Right	15 (19%)		38 (33%)		43 (33%)		10 (16%)		11 (25%)		4 (12%)		32 (38%)		6 (20%)	
**IVC**																
Interrupted	0 (0%)		114 (99%)	0.0001	84 (65%)		30 (48%)	0.02	0 (0%)		0 (0%)	-	84 (99%)		30 (100%)	0.35
Normal	77(100%)		1 (1%)		45 (35%)		33 (52%)		44(100%)		33 (100%)		1 (1%)		0 (0%)	

Table S4:

Associated congenital heart diseases in different groups

Groups n= 192	RAI n=77 (%)	vs	LAI n=115(%)	P	D-hand n=129(%)	vs	L-hand n=63(%)	P	D-hand in RAI n=44(%)	vs	L-hand in RAI n=33(%)	P	D-hand in LAI n=85(%)	vs	L-hand in LAI n=30(%)	P
**PVD**	42 (54%)		37 (32%)	0.0001	54 (42%)		25 (40%)	0.39	25 (57%)		17 (51%)	0.89	29 (34%)		8 (27%)	0.59
PAPVD	7 (9%)		27 (23%)		26 (20%)		8 (13%)		4 (9%)		3 (9%)		22 (26%)		5 (17%)	
TAPVD	35 (45%)		10 (9%)		28 (22%)		17 (27%)		21 (48%)		14 (42%)		7 (8%)		3 (10%)	
**AVSD**	68 (88%)		49 (43%)	0.0001	76 (59%)		41 (65%)	0.43	40 (91%)		28 (85)	0.16	36 (42%)		13 (43%)	0.71
Balanced	8 (10%)		18 (16%)		19 (15%)		7 (11%)		7 (16%)		1 (3%)		12 (14%)		6 (20%)	
Unbalanced	60 (78%)		31 (27%)		57 (44%)		34 (54%)		33 (75%)		27 (82%)		24 (28%)		7 (23%)	
**FUVH**	63 (82%)		44 (38%)	0.0001	67 (52%)		40 (64%)	0.31	34 (78%)		29 (87%)	0.43	33 (39%)		11 (37%)	0.63
d-LV	26 (34%)		13 (11%)		24 (19%)		15 (24%)		13 (30%)		13 (39%)		11 (13%)		2 (7%)	
d-RV	37 (48%)		31 (27%)		43 (33%)		25 (40%)		21(48%)		16 (48%)		22 (26%)		9 (30%)	
**PV**	72 (93%)		64 (56%)	0.0001	88 (68%)		48 (77%)	0.37	40 (91%)		32 (97%)	0.55	48 (56%)		16 (53%)	0.85
PA	28 (36%)		17 (15%)		27 (21%)		18 (29%)		15 (34%)		13 (39%)		12 (14%)		5 (17%)	
PS	44 (57%)		47 (41%)		61 (47%)		30 (48%)		25 (57%)		19 (58%)		36 (42%)		11 (36%)	
**TGA**	29 (38%)		18 (16%)	0.001	29 (22%)		18 (29%)	0.36	17 (39%)		12 (36%)	0.84	12 (14%)		6 (20%)	0.45
**DORV**	36 (47%)		30 (26%)	0.003	48 (37%)		18 (29%)	0.24	24 (55%)		12 (36%)	0.11	24 (28%)		6 (20%)	0.38
**AAA**	2 (3%)		9 (8%)	0.13	10 (8%)		1 (2%)	0.08	2 (5%)		0 (0%)	0.22	8 (9%)		1 (3%)	0.29
**HLHS**	0 (0%)		4 (3%)	0.098	3 (2%)		1 (2%)	0.74	0 (0%)		0 (0%)	-	3 (4%)		1 (3%)	0.96
**CA**	20 (26%)		29 (25%)	0.91	33 (26%)		16 (25%)	0.98	14 (32%)		6 (18%)	0.18	19 (22%)		10 (33%)	0.23

Table S5:

Congenital disorders of excitation and conduction in different groups

Groups n= 192	RAI n=77 (%)	vs	LAI n=115(%)	P	D-hand n=129(%)	vs	L-hand n=63(%)	P	D-hand in RAI n=44(%)	vs	L-hand in RAI n=33(%)	P	D-hand in LAI n=85(%)	vs	L-hand in LAI n=30(%)	P
**No ECG**	4 (5 %)		1 (1 %)		2 (2 %)		3 (5 %)		1 (2 %)		3 (9 %)		1 (1 %)		0 (0 %)	
**P wave axis**				0.0001				0.0001				0.04				0.001
LIA	36 (47%)		38 (33%)		57 (44%)		17 (27%)		25 (57%)		11 (33%)		32 (38%)		6 (20%)	
LSA	2 (3%)		39 (34%)		36 (28%)		5 (8%)		2 (4 %)		0 (0%)		34 (40%)		5 (17%)	
RIA	32 (41%)		19 (17%)		24 (18%)		27 (43%)		16 (36%)		16 (48%)		8 (9%)		11 (37%)	
RSA	3 (4%)		18 (15%)		10 (8%)		11 (17%)		0 (0%)		3 (9%)		10 (12%)		8 (27%)	
**P wave axis combined**																
**Left axis**	38 (50%)		77 (67%)	0.02	93 (72%)		22 (35%)	0.0001	27 (61%)		11 (33%)	0.04	66 (78%)		11 (37%)	0.0001
**Right axis**	35 (45%)		37 (32%)		34 (26%)		38 (60%)		16 (36%)		19 (58%)		18 (21%)		19 (63%)	
**Superior axis**	5 (7%)		57 (50%)	0.0001	46 (35%)		16 (25%)	0.185	2 (5%)		3 (9%)	0.28	44 (52%)		13 (43%)	0.58
**Inferior axis**	68 (88%)		57 (50%)		81 (63%)		44 (70%)		41 (93%)		27 (81%)		40 (47%)		17 (57%)	
**Atrial Arrhythmias**	18 (23%)		26 (23%)	0.175	30 (23%)		14 (22%)	0.42	12 (27%)		6 (18%)	0.31	18 (21%)		8 (27%)	0.71
**Conduction defects (total)**	10 (13%)		41 (35%)	0.003	33 (25%)		18 (28%)	0.21	6 (14%)		4 (12%)	0.33	27 (31%)		14 (47%)	0.18
**AV block (total)**	1 (1%)		4 (3%)		1 (1%)		4 (6%)		0 (0%)		1 (3%)		1 (1%)		3 (10%)	
CHB-c	0 (0%)		2 (2%)		0 (0%)		2 (3%)		0 (0%)		0 (0%)		0 (0%)		2 (7%)	
CHB-po	1 (1%)		0 (0%)		0 (0%)		1 (2%)		0 (0%)		1 (3%)		0 (0%)		0 (0%)	
CHB-acquired	0 (0%)		2 (2%)		1 (1%)		1 (2%)		0 (0%)		0 (0%)		1 (1%)		1 (3%)	
**Junctional rhythm (total)**	9 (12%)		37 (32%)		32 (24%)		14 (22%)		6 (14%)		3 (9%)		26 (30%)		11 (37%)	
JIR	9 (12%)		27 (23%)		25 (19%)		11 (17%)		6 (14%)		3 (9%)		19 (22%)		8 (27%)	
JPR	0 (0%)		10 (9%)		7 (5%)		3 (5%)		0 (0%)		0 (0%)		7 (8%)		3 (10%)	
**Ventricular arrhythmias**	1 (1%)		1 (1%)	0.17	2 (2%)		0 (0%)	0.26	1 (2%)		0 (0%)	0.29	1 (1%)		0 (0%)	0.7
**PPM**	2 (3%)		16 (14%)	0.008	10 (8%)		8 (13%)	0.27	1 (2%)		1 (3%)	0.84	9 (11%)		7 (23%)	0.08

Table S6:

Mortality in different groups

Groups n= 192	RAI n=77 (%)	vs	LAI n=115(%)	P	D-hand n=129(%)	vs	L-hand n=63(%)	P	D-hand in RAI n=44(%)	vs	L-hand in RAI n=33(%)	P	D-hand in LAI n=85(%)	vs	L-hand in LAI n=30(%)	P
**Dead n (%)**	26 (34%)		21 (18%)	0.0001	32 (25%)		15 (24%)	0.53	14 (32%)		12 (36%)	0.86	18 (21%)		3 (10%)	0.07
**Alive n (%)**	35 (45%)		87 (76%)		80 (62%)		42 (67%)		20 (45%)		15 (45%)		60 (71%)		27 (90%)	
**Missed n (%)**	16 (21%)		7 (6%)		17 (31%)		6 (10%)		10 (23%)		6 (18%)		7 (8%)		0 (0%)	

Table S7:

Survival at 5 years, 10 years and 15 years, respectively in different groups

Groups n= 192	RAI n=77 (%)	vs	LAI n=115(%)	P	D-hand n=129(%)	vs	L-hand n=63(%)	P	D-hand in RAI n=44(%)	vs	L-hand in RAI n=33(%)	P	D-hand in LAI n=85(%)	vs	L-hand in LAI n=30(%)	P
**5 Years**	65 %		88 %	0.0001	78 %		78 %	0.87	68 %		61 %	0.46	84 %		97 %	0.08
**10 Years**	65 %		83 %		75 %		78 %		68 %		61 %		84 %		97 %	
**15 Years**	56 %		79 %		67 %		56 %		55 %		61 %		72 %		97 %	
